# Teaching Minimally Invasive Interventions in Paediatric Dentistry: A Cross-Sectional Survey of Dental Schools in Iran

**DOI:** 10.1186/s12903-021-01735-5

**Published:** 2021-07-23

**Authors:** S. Moradi, S. Sabbagh, L. Timms, V. Ravaghi

**Affiliations:** 1 Dental Research Center, Research Institute of Dental Sciences, Shahid Beheshti University of Medical Sciences, Tehran, Iran; 2 Dental Materials Research Center, Mashhad University of Medical Sciences, Mashhad, Iran; 3 School of Clinical Dentistry, The University of Sheffield, Sheffield, UK; 4 School of Dentistry, University of Birmingham, Birmingham, UK

**Keywords:** Paediatric dentistry, Minimal intervention dentistry, Cariostatic agents, Dental caries, Silver diamine fluoride, Hall technique

## Abstract

**Background:**

Dental caries is a significant public health problem in Iran. Teaching  minimally invasive interventions in paediatric dentistry may facilitate the provision of treatment for untreated dental caries in children. We evaluated the teaching of such interventions in both undergraduate dental curriculum and Paediatric Dentistry Specialty Training Programme (PDSTP) in Iran.

**Methods:**

This was a cross-sectional questionnaire-based survey. Participants in this study were the directors of 40 undergraduate programmes and 15 PDSTPs in all Iranian dental schools (response rate = 100%). Descriptive statistics were reported.

**Results:**

The most commonly taught methods were preventive fissure sealant and preventive resin restoration (PRR), which were taught ‘both didactically and clinically’ in all undergraduate dental programmes. The least commonly taught methods were silver diamine fluoride (SDF), the Hall technique and resin infiltration, which were taught ‘both didactically and clinically’ in less than 5% of dental schools. The same three methods were the least commonly approaches taught in PDSTP, further, they were less often perceived to be ‘essential’.

**Conclusions:**

There was a notable variation in the teaching of the management of dental caries in Iran’s dental education. Some minimally invasive approaches including SDF, the Hall technique and resin infiltration are not being commonly taught in Iranian dental schools despite the evidence base for these techniques.

## Background

Dental caries was historically believed to be an infectious disease and thus dental professionals based treatment on the principle that removal of all infected carious tooth tissue was required to halt caries progression [1]. In the light of recent advances in cariology, there has been a paradigm shift in the management of dental caries from the non-selective complete removal of infected tissue to more minimally invasive interventions. Dental caries is now regarded as a disease, which originates from the imbalance of demineralisation and remineralisation of tooth structure as a result of metabolism from the plaque biofilm and propagated by dietary sugars. As such, the management of them is focussed on controlling the biofilm and cariogenic environment that leads to demineralisation rather than elimination of infected tissue [2]. In addition, a number of limitations are attached to using conventional non-selective methods for management of carious lesions. Removing demineralised, yet structurally intact dentine, weakens tooth structure [3] and increases the risk of iatrogenic pulpal exposure [4]. Using conventional methods are also associated with more pain and discomfort [5], therefore, they may trigger dental anxiety and discourage interest in future dental visits. In addition, using MID techniques for children with severe dental anxiety may potentially prevent extraction under general anaesthetic.

Minimal intervention dentistry (MID) is defined as ‘a holistic management philosophy that integrates carious lesion control and minimal operative intervention’. In recent years, there has been a renewed interest in application of MID in management of carious lesions especially for primary dentition. Emergence of several systematic reviews to summarise the evidence for effectiveness of silver diamine fluoride (SDF) [6] and investigating the effectiveness of minimally invasive biological techniques in a multicentre UK based clinical trial (known as FICTION study) [7] are indicative of such interest.

Significant reduction of dental caries in children is documented in developed countries such as the UK over the past decades [8]. In contrast, it is believed that prevalence of childhood dental caries is growing rapidly in the less affluent low and middle-income countries (LMICs), mainly due to change in lifestyle and diet habits [9]. Among other reasons, conventional approaches for management of dental caries may hamper the delivery of dental care in less affluent countries as they require costly and advanced high speed technologies [1]; the environment which may not be always available in lower and middle income countries [10]. Furthermore, following the COVID pandemic and given to concerns about transmission of coronavirus through Aerosol Generating Procedures (AGPs), there has been a new drive for adoption of the AGP-free interventions of MID in different dental specialities. In paediatric dental care specifically, there have been calls to utilise the potentials of MID to facilitate dental care in the post COVID era [11].

Iran is an upper–middle-income country with relatively high number of dentist per capita (3.3 dentists for 10,000 population), however, there are major issues with uneven distribution of the dental work force in Iran [12]. In the latest dental surveys of children, the prevalence of dental caries among pre-school children in Iran was high (decayed, missing and filled teeth (dmft) = 5.84) [13]. Teaching minimal intervention dentistry for management of dental caries in Iranian dental schools can pave the way for its wider application in primary care setting to tackle childhood dental caries. Teaching and application of techniques used for management of dental caries have been previously studied in several countries such as Canada and the US [14–17].

This study aims to evaluate the teaching of managing childhood dental caries in both undergraduate dental curriculum and PDSPTs in Iran. We also examine the opinions of dental educators toward the relevance of teaching each method in their programme.

## Methods

This is a cross-sectional questionnaire-based survey, which was carried out in the summer of 2020. This study aims to evaluate and compare the teaching of management of dental caries in undergraduate curriculum and PDSTP in all dental schools (n = 40) in Iran. Directors of 40 undergraduate programmes and 15 PDSTPs were invited to take part in this study by completing the self-administered questionnaires. The purpose of the study was clearly outlined in the information sheet. The ethical approval for this study was obtained from Shahid Beheshti University of Medical Sciences, Iran.

The study questionnaire consists of two sections. We asked participants to report which minimally invasive methods for management of dental caries or carious lesions (for both primary and permanent dentitions) are being taught in their corresponding programmes. Respondents could choose whether the teaching of each intervention was covered in ‘didactical’  or in ‘clinical’ training. Respondents were informed that clinical training should be indicated only if the students carry out the treatment in patient clinics. The information sheet also explained that the teaching sessions, which involved practicing on phantom heads and observing the delivery of treatment, should be regarded as ‘didactical’ training. We also asked directors of programmes to report whether they believe the clinical training of each method were relevant for their programmes; to which they could respond essential/ not essential or no comment. The questionnaire was administered in Farsi, which is the only language of instruction in Iran.

Developing a questionnaire for this study was guided by the terminology and framework, which has been proposed by the International Caries Consensus Collaboration (ICCC) [14]. Incorporating the categories in this framework, we identified a number of interventions for managing dental caries and carious lesions following consultation with paediatrics dentists and dental public health academics. To ensure the content validity, this list was reviewed and finally thirteen techniques were agreed upon by three members of research team (SM, VR, and SS). Patient self-care (e.g. Toothbrushing instruction) and the techniques based on complete removal of affected tissues were not included. The questionnaire was then sent to participants using a web-based survey tool (i.e. Google forms). We sent reminders to participants who did not complete the survey in weekly intervals. Descriptive statistics (frequencies and percentages) were reported, but no statistical test was carried out in this research. Test–retest reliability of the questionnaire was examined by asking five participants to answer to the questionnaire two weeks after first completion. The test–retest reliability was indicated by 77.6% agreement between the first and second responses to items.

## Results

All directors of undergraduate programmes (n = 40) and PDSTPs (n = 15) in Iranian dental schools agreed and participated in this study, therefore, we achieved the response rate of 100%. Furthermore, all questions were answered in all returned questionnaires, which means this study had no missing data.

Tables 1 and 2 report the teaching of managing dental caries and carious lesions in undergraduate programmes and PDSTPs, respectively. These tables also report whether the programme directors perceive the clinical teaching of these interventions as ‘essential’. According to Table 1, the most commonly taught methods in undergraduate programmes were preventive fissure sealant and preventive resin restoration (PRR). These interventions were taught ‘both didactically and clinically’ in all dental schools. On the other hand, the least commonly taught method was the use of SDF; with no undergraduate programme reporting ‘both didactic and clinical’ teaching. Despite this, nearly half of undergraduate programmes (42.5%) reported the didactic teaching of SDF. After SDF, the Hall technique and resin infiltration were the least commonly taught methods with only 5% of undergraduate dental programmes teaching both didactically and clinically. The same three management methods (i.e. SDF, Hall technique, and resin infiltration) were least often perceived to be ‘essential’ by the directors of undergraduate dental programmes. For example, the Hall technique was perceived to be essential by only one out five (20%) directors of undergraduate programmes.

**Table 1:** Teaching management of dental caries and carious lesions in undergraduate-curriculum in Iran (n = 40)

	Teaching status % (N)				Perception of programme directors with regard to clinical training % (N)		
	Didactically only	Clinically only	Both didactically and clinically	None	Essential	Not essential	No comment
Fluoride varnish	7.5 (3)	0	92.5 (37)	0	100 (40)	0	0
Fluoride gel	17.5 (7)	0	77.5 (31)	5 (2)	90 (36)	5 (2)	5 (2)
Preventive fissure sealant	0	0	100 (40)	0	100 (40)	0	0
Therapeutic sealant	2.5 (1)	0	82.5 (33)	15 (6)	87.5 (35)	7.5 (3)	5 (2)
Resin infiltration	50 (20)	2.5 (1)	5 (2)	42.5 (17)	25 (10)	50 (20)	25 (10)
Atraumatic restorative treatment	50 (20)	0	45 (18)	5 (2)	65 (26)	20 (8)	15 (6)
Preventive resin restoration	0	0	100 (40)	0	100 (40)	0	0
Silver diamine fluoride	42.5 (17)	2.5 (1)	0	55 (22)	52.5 (21)	22.5 (9)	25 (10)
Hall-technique	52.5 (21)	0	5 (2)	42.5 (17)	20 (8)	67.5 (27)	12.5 (5)
Interim therapeutic restoration	47.5 (19)	0	30 (12)	22.5 (9)	65 (26)	17.5 (7)	17.5 (7)
Indirect pulp capping	12.5 (5)	0	87.5 (35)	0	97.5 (39)	2.5 (1)	0
Making carious lesion cleansable	42.5 (17)	0	17.5 (7)	40 (16)	62.5 (25)	10 (4)	27.5 (11)
Stepwise caries removal	50 (20)	0	40 (16)	10 (4)	72.5 (29)	6 (15)	12.5 (5)

**Table 2:** Teaching management of dental caries and carious lesions in PDSTPs in Iran (n = 15)

	Teaching status % (N)				Perception of programme directors with regard to clinical training % (N)		
	Didactically only	Clinically only	Both didactically and clinically	None	Essential	Not Essential	No comment
Fluoride varnish	0	0	100 (15)	0	100 (15)	0	0
Fluoride gel	20 (3)	6.7 (1)	73.3 (11)	0	86.7 (13)	6.7 (1)	6.7 (1)
Preventive fissure sealant	0	0	100 (15)	0	93.3 (14)	6.7 (1)	0
Therapeutic sealant	13.3 (2)	0	80 (12)	6.7 (1)	93.3 (14)	0	6.7 (1)
Resin infiltration	60 (9)	0	33.3 (5)	6.7 (1)	93.3 (14)	0	6.7 (1)
Atraumatic restorative treatment	40 (6)	0	60 (9)	0	93.3 (14)	6.7 (1)	0
Preventive resin restoration	0	0	100 (15)	0	100 (15)	0	0
Silver diamine fluoride	73.3 (11)	6.7 (1)	6.7 (1)	13.3 (2)	80 (12)	0	20 (3)
Hall-technique	60 (9)	0	20 (3)	20 (3)	53.3 (8)	26.7 (4)	20 (3)
Interim therapeutic restoration	40 (6)	6.7 (1)	53.3 (8)	0	86.7 (13)	0	13.3 (2)
Indirect pulp capping	0	0	100 (15)	0	93.3 (14)	0	6.7 (1)
Making carious lesion cleansable	20 (3)	13.3 (2)	40 (6)	26.7 (4)	66.7 (10)	6.7 (1)	26.7 (4)
Stepwise caries removal	20 (3)	0	73.3 (11)	6.7 (1)	93.3 (14)	6.7 (1)	0

According to Table 2, some management methods such as fluoride varnish, and PRR were taught in all PDSTPs. In contrast, the Hall technique, SDF and resin infiltration were the least commonly taught methods in PDSTPs, which replicated the findings of undergraduate programmes. The SDF, for example, was being taught ‘both didactically and clinically’ in only one out of fifteen programmes (6.7%). All three aforementioned methods were taught ‘didactically only’ in majority of PDSTPs.

Interestingly, in both undergraduate and PDSTPs, some techniques were reportedly taught only clinically in some schools. For example, the SDF and resin infiltration were taught ‘clinically only’ in one undergraduate dental programme.

## Discussion

This study demonstrates a notable variation in the teaching of minimally invasive interventions for the management of dental caries and carious lesions in Iran’s dental education. While some techniques such as preventive fissure sealant, PRR, and fluoride varnish are widely taught across dental programmes, three methods received little attention. These methods were SDF, the Hall technique, and resin infiltration. SDF, for example, was not taught ‘both didactically and clinically’ in any undergraduate dental programme followed by the Hall technique and resin infiltration, which were taught by only two out of forty undergraduate dental programmes. A similar pattern was observed for PDSTPs with the same three approaches (i.e. SDF, Hall technique, and resin infiltration) being less commonly taught ‘both didactically and clinically’.

The cross-sectional nature of this study did not allow us to explore the underlying reasons for variation in adoption of minimally invasive techniques. We are, nevertheless, able to propose some likely scenarios as to why certain techniques such as SDF and the Hall technique were not incorporated in clinical setting. Given that SDF and the Hall technique were widely taught didactically, it is evident that these approaches are known to Iranian dental educators. However, when we queried the opinions of directors of dental programmes, it became apparent that these methods were largely deemed not ‘essential’. It can be argued that the lower popularity of these approaches in clinical setting reflects the lack of confidence in their effectiveness or safety among Iranian dental educators. This argument appears sensible given that the evidence for effectiveness and safety of these two approaches has fairly recently made available as compared to well-established approaches such as application of fluoride varnish and fissure sealants. On a different note, the decision to incorporate the clinical teaching of these interventions are influenced by external factors such as parental acceptance of the methods, their cost, availability of materials in the market and the clinical competence of the educator. Future studies may explore the barriers for underutilising these techniques in Iran’s dental education.

The lower adoption of some techniques such SDF and Hall technique in Iranian dental education is in contrast with the increasing popularity of these techniques in the US dental education. Majority of US undergraduate dental programmes reported the teaching of SDF in 2018 [18]. The use of SDF in the clinical setting was reported by only a quarter of graduate dental programmes in the US in 2015 [19]; the rate which has increased to 100% in 2020 [20], most probably due to publication of guideline for the use of SDF by the American Academy of Pediatric Dentistry [21].

Hall technique is a relatively new technique of placing stainless steel crowns and biologically seals carious lesions in primary molars which pauses or slows down the progression of caries to allow the primary tooth exfoliate before causing pain [22]. Over the past decade, a wealth of evidence emerged to support its clinical effectiveness and cost-effectiveness of Hall technique [7, 23–25].

SDF is becoming increasingly recognised as an appropriate method for arresting carious lesions and to prevent progression of cavitated caries. A recent umbrella review indicates that application of 38% SDF can successfully arrest non-cavitated carious lesions in enamel and dentin of primary teeth. It can also be used to manage extensive and non-restorable carious lesions without pain/infection until tooth exfoliation occurs [6]. There has been an increasing interest to adopt this new method in dental practices in developed countries such as the UK [26] particularly given its relatively easy application [27].

Resin infiltration has recently gained more acceptance as a method of arresting and controlling proximal non-cavitated carious lesions extending radiographically up to the outer third of dentine [28]. A recent meta-analysis indicated the effectiveness of this approach in permanent teeth; nevertheless, no conclusive evidence exists to support resin infiltration application in primary teeth as well as its longevity [29].

This study highlighted a significant gap in the teaching of managing dental caries in Iranian dental education. This research showed that some of the contemporary; yet effective, methods of managing carious lesions such as SDF and Hall technique received little attention in both undergraduate programmes and PDSTPs. Given the magnitude of dental caries problem in Iran, revising the training of dental professionals to include these underutilised methods is fundamental to delivery of dental care in Iran. In Iran, general dental practitioners and specialist paediatric dentists largely remain the sole providers of dental care for children. Educating dental providers to use less invasive methods for managing dental caries not only improves clinical patient outcomes, but also may pave the way for better access to dental care within primary care due to reduced cost and time of the treatment.

In terms of global impacts, introducing MID for the management of dental caries can bring additional benefit to delivery of dental care in less affluent countries and LMICs. Conventional techniques, which rely on complete removal of affected tissues, may only be carried out by highly skilled workforces (i.e. dentists using advanced equipment). We, however, recognise that many LMICs face scarcity of trained dental professionals. For example, it is reported that 93% of WHO member states have less than 1 dentistry personnel per 1000 population [30]. Wider application of minimally invasive approaches, therefore, can be viewed as an opportunity for public health programmes in LMICs. In line with this, the 2016 Basic Package of Oral Care by the WHO Regional Office for Africa [10] advised the use of an established minimally invasive approach known as atraumatic restorative treatment (ART). More recently, the WHO guidance on the WHO implementation manual ‘Ending Childhood Dental Caries’ endorsed the use of minimally invasive techniques such as SDF and ART [31].

This study had a number of strengths and limitations. Participation of directors of programmes from all dental schools as well the excellent response rate of 100% in our survey was one of the strengths of this study. Prior to administering the questionnaire and in order to achieve the best response rate, we introduced the project and sought advice from a number of nationally known dental leaders who had previously held positions in local and national dental associations. The informal briefing of our research project may have contributed to achieving the desirable response rate. One may also argue that the directors of programmes appreciated the relevance of this study and therefore were keen to participate. Further, due to the online survey that prohibited submission without completion of all questions, the returning questionnaires yielded no missing data. Several measures were also taken to ensure the appropriateness of the terminology used in this questionnaire. Although a number of languages are spoken in Iran, Farsi is the only official language of instruction; therefore, the questionnaires were administered in Farsi. To enhance the accuracy of translation, after selection of the interventions, we sought advice from specialist paediatric dentists to ensure that the terminology used in the questionnaire matches with those of translated paediatric dentistry textbooks being taught in Iran. This highlights one of the practical challenges of designing a research project in geographies and localities where the language of instruction is not English. This study had a number of limitations. Generally, there is a lack of agreement with regard to describing dental interventions as minimally invasive, which also applies to this study. This study collected data from directors of paediatric dentistry programmes. It is likely that some of the methods we questioned in this study are being taught in other parts of the curriculum such as restorative dentistry. We contemplated the idea of sending the questionnaire to directors of dental educations in each school to address this concern. Following the consultation with the local gatekeepers, however, this option was not pursued for pragmatic reasons and in order to ensure an acceptable response rate. Furthermore, small number of programmes reported teaching some methods ‘clinically only’. While this may sound justifiable for PDSTPs, it is unclear to us how some undergraduate programmes may have taught some techniques clinically only without prior didactic background. Incorrect responses and lack of clarity could be other reasons explaining these unexpected findings. Finally, we used closed-ended questionnaire to investigate the perception of dental educators. Qualitative study design is needed to obtain an in-depth understanding of the barriers, which led to lower adoption of some management methods such as SDF; also to understand the variation in the perception of programme directors. Finally yet importantly, the minimally invasive interventions that we included in this questionnaire were collated after extensive consultation with the experts and following comprehensive review of relevant literature. Despite this, the arbitrary approach toward creating the list may be considered as a limitation.

## Conclusion

Teaching of some evidence-based management methods of dental caries in children has been limited in Iran’s dental education. Future studies may identify the barriers to adoption of these approaches.

## Abbreviations

PDSTP: Paediatric Dentistry Specialty Training Programme
PRR: Preventive resin restoration
SDF: Silver diamine fluoride
MID: Minimal intervention dentistry
LMICs: Low and middle-income countries
AGPs: Aerosol generating procedures
dmft: Decayed, missing and filled (primary) teeth
ICCC: International Caries Consensus Collaboration
ART: Atraumatic restorative treatment
